# Large-Area Growth of Turbostratic Graphene on Ni(111) via Physical Vapor Deposition

**DOI:** 10.1038/srep19804

**Published:** 2016-01-29

**Authors:** Joseph A. Garlow, Lawrence K. Barrett, Lijun Wu, Kim Kisslinger, Yimei Zhu, Javier F. Pulecio

**Affiliations:** 1 Condensed Matter Physics and Material Science Department, Brookhaven National Laboratory, Upton, NY 11973; 2 Material Science and Engineering Department, Stony Brook University, Stony Brook, NY 11794; 3 Division of Materials Science and Engineering, Boston University, Boston, MA 02215; 4 Center for Functional Nanomaterials, Brookhaven National Laboratory, Upton, NY 11973.

## Abstract

Single-layer graphene has demonstrated remarkable electronic properties that are strongly influenced by interfacial bonding and break down for the lowest energy configuration of stacked graphene layers (AB Bernal). Multilayer graphene with relative rotations between carbon layers, known as turbostratic graphene, can effectively decouple the electronic states of adjacent layers, preserving properties similar to that of SLG. While the growth of AB Bernal graphene through chemical vapor deposition has been widely reported, we investigate the growth of turbostratic graphene on heteroepitaxial Ni(111) thin films utilizing physical vapor deposition. By varying the carbon deposition temperature between 800 –1100 °C, we report an increase in the graphene quality concomitant with a transition in the size of uniform thickness graphene, ranging from nanocrystallites to thousands of square microns. Combination Raman modes of as-grown graphene within the frequency range of 1650 cm^−1^ to 2300 cm^−1^, along with features of the Raman 2D mode, were employed as signatures of turbostratic graphene. Bilayer and multilayer graphene were directly identified from areas that exhibited Raman characteristics of turbostratic graphene using high-resolution TEM imaging. Raman maps of the pertinent modes reveal large regions of turbostratic graphene on Ni(111) thin films at a deposition temperature of 1100 °C.

Graphene synthesis originated in the 1960s with graphitization of SiC^1^ as well as through chemical vapor deposition (CVD) on Pt(111)^2^. It was not until Novoselov and Giem established micromechanical cleavage of bulk graphite as a repeatable method to isolate single-layer graphene (SLG)^3^ that graphene began to receive tremendous interest from academia and industry^4^. To date, graphene has been fabricated from a range of carbon sources through a variety of top-down and bottom-up approaches including, mechanical and chemical exfoliation of graphite^5^, CVD^6^,^7^, molecular beam epitaxy^8^,^9^, graphitization of SiC^10^, longitudinal unzipping of carbon nanotubes^11^ and, growth from solid carbon sources, such as polymers^12^. Widely considered a prominent growth technique, CVD has produced graphene on the meter scale^13^, and through precise constraint of growth parameters, has demonstrated control over the morphology^14^. Despite these notable advances, out-of-plane π-orbital hybridization between SLG and its substrate^15^ can limit its theoretical capacity due to charge scattering^16^.

SLG is a two-dimensional network of sp^2^ bonded carbon atoms that exhibits near-ballistic transport of electrons^17^, among other remarkable properties^18^, enabling a broad range of applications from graphene field effect transistors^19^ for carbon-based electronics to molecular sieves^20^ for water treatment. As a true two-dimensional system, graphene provides an intriguing opportunity to study the fundamental surface physics and interfacial interactions and, in turn, their property/function relations. For example, recent graphene studies have focused on the impact of interfacial interactions on charge transportation^17^ and spin injection efficiency^21^. It is known that interfacial interactions between graphene and its substrate drastically alter the electronic structure of SLG^15^. Moreover, interlayer coupling between adjacent carbon layers in multilayer graphene (MLG) also modifies the dispersion of electronic states and can open a band-gap^22^, suppressing desirable properties of SLG.

Relative rotations between graphene layers could provide a novel route to overcome the restrictions interfacial interactions impose on the desirable electronic properties of SLG. Relative rotations between adjacent graphene layers can alleviate π-orbital hybridization, thus, restoring the electronic structure of SLG in a MLG configuration^23^. A schematic illustration of layered graphene films with and without relative layer rotations can be seen in Fig. 1. The honeycomb lattice of graphene is composed of two overlapping triangular sublattices, A (orange highlight) and B (blue highlight). For AB Bernal stacked bilayer graphene (BLG), the B site of the top layer sits directly above the A site of the bottom layer leading to electronic coupling between the two layers as shown in the cross-sectional view of Fig. 1a. Figure 1b shows a similar bilayer where the top layer is rotated 20° about the central AB site. Rotational stacking mitigates interlayer coupling, increasing interplanar spacing, and produces unique phonon based Raman spectral signatures^24^,^25^. In fact, MLG with a relative rotation between layers greater than 3° maintains linear dispersion of the valence and conduction bands in the low energy regime with a renormalized Fermi velocity, whereas rotations above 20° effectively decouple layers, preserving the unique electronic states of SLG^26^. Thus, MLG with stacking order that does not subdue the electronic properties of SLG could enable the ballistic transport of electrons through decoupled graphene layers.

Turbostratic graphene is comprised of multiple graphene layers with interlayer rotations that alleviate orbital hybridization resulting in carrier mobilities similar to that of SLG. Its relevance has been limited by the difficulties associated with its controlled and reproducible growth, along with its quantitative characterization. Turbostratic graphene has been grown tens of layers thick on SiC and observed through Moiré patterns using STM^27^,^28^,^29^, though its integration onto other substrates presents challenges. Additionally, localized areas of twisted bilayer and few-layer graphene have been investigated using characteristic Raman signatures after growth through CVD on Cu or by manipulating mechanically exfoliated graphene sheets^30^,^31^,^32^,^33^,^34^,^35^,^36^,^37^. While these findings are motivating, the properties of as-grown turbostratic graphene and its possible application remain largely unexplored. Here, heteroepitaxial Ni(111) thin films were grown on magnesium oxide (MgO(111))^38^,^39^ followed by the direct deposition of carbon at substrate temperatures 800 °C, 900 °C, 1000 °C and 1100 °C, in a continuous process via physical vapor deposition (PVD) (see Methods section). We systematically investigate the prevalence of as-grown turbostratic graphene with combination Raman modes within the region 1650–2300 cm^−1^ 30,31, various features of the 2D peak^25^,^40^ and electron microscopy.

## Results and Discussion

### Graphene growth on Ni(111)

**Figure 2:**
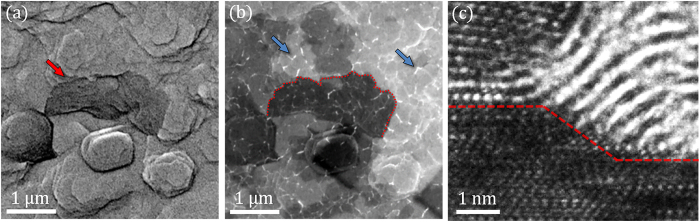
Graphene growth on Ni(111). (**a**) An SEM image, obtained with an in-lens detector, reveals the surface morphology of the continuous nickel thin film composed of steps and terraces post-graphene growth at 1000 °C. The red arrow shows a prominent step edge. (**b**) An SEM image of the same region shown in (**a**), taken with a low angle secondary electron detector that is more sensitive to the graphene layers. The red outline serves to show the same step-edge depicted in (**a**). The areas of dark contrast correspond to thicker graphene layers due to attenuation of the secondary electrons emitted from the underlying nickel. The nickel thin film is entirely covered with graphene as evidenced by the presence of graphene wrinkles, which appear as bright contrast (blue arrows). (**c**) A cross-sectional TEM image that exemplifies a graphene growth mechanism on nickel. The red line shows the nickel and graphene interface where the dark region underneath is the nickel substrate. The faceted step edge serves as a nucleation region for thicker multilayer graphene while the higher terrace is covered by bilayer graphene.

Graphene has been grown on a variety of transition metal surfaces such as Co(0001), Cu(111), Pd(111), Pt(111), Ir(111), Ru(0001), Ni(111) and polycrystalline nickel. Single crystal substrates are advantageous growth templates as they increase homogeneity of the grown graphene films through a reduction in nucleation sites^41^,^42^. The Ni(111) lattice plane is a desirable graphene substrate due to its close lattice match with graphene; the lattice parameter of graphene is 246 pm while Ni(111) has a lattice parameter of 249 pm. The surface morphology of Ni(111) thin films is comprised of steps and terraces^39^, as shown in Fig. 2a. The growth of graphene on nickel at high temperatures is a dynamic process involving a combination of sub-surface atomic interactions and nucleation mechanisms^43^,^44^. Carbon diffuses into the heated nickel substrate then graphene nucleates on the nickel surface through precipitation at step-edges, and segregation on terraces. Graphene precipitation refers to a carbon phase separation and occurs out of the carbon-nickel solution at step-edges during cooling^43^, meanwhile, a segregation phase exists where graphene forms on the nickel terraces below the solubility limit^43^. Figure 2b portrays a graphene patch that nucleated at a nickel step-edge, likely through precipitation during cooling^45^,^46^. Direct evidence of the relationship between graphene and the nickel step-edge is observed from the TEM image shown in Fig. 2c. It reveals a multilayer graphene domain that nucleated at the nickel step-edge from a cross-sectional perspective and exemplifies a growth mechanism of graphene on nickel. The carbon layers remain bonded to the nickel step-edge, demonstrating their strong interaction. Interestingly, the image shows a bilayer graphene region on top of the terrace (left) which overlays the multilayer domain (right), potentially contributing to the formation of rotationally stacked graphene layers. Graphene precipitation at step-edges is energetically favorable to formation on nickel terraces, inherently generating graphene film heterogeneity due to difference in nucleation rates between the two regions. The deposition temperature and chemical potential critically impact the nucleation rates at the step-edge and at the terrace, modulating graphene film morphology^46^.

### Temperature dependent PVD graphene growth

Of recent interest, particularly for deposition on insulating, magnetic and ferroelectric substrates^8^, PVD allows direct deposition onto a desired substrate through sublimation of a graphitic source. PVD at low pressures is an advantageous growth technique that enables line-of-sight deposition with real-time sub-angstrom per second measurement of the atomic carbon flux, in addition to precise control over the quantity of deposited material. Further, it permits direct control over sample temperature and allows the growth of complex heterostructures without breaking vacuum. Specifically for the growth of graphene on carbon-soluble substrates, PVD supports modulation of carbon saturation, the saturation rate, post-deposition annealing and sample cooling rates. Furthermore, it can be tailored to grow graphene directly on a substrate of interest, enabling top-down fabrication techniques to employ as-grown samples in device configurations.

**Figure 3:**
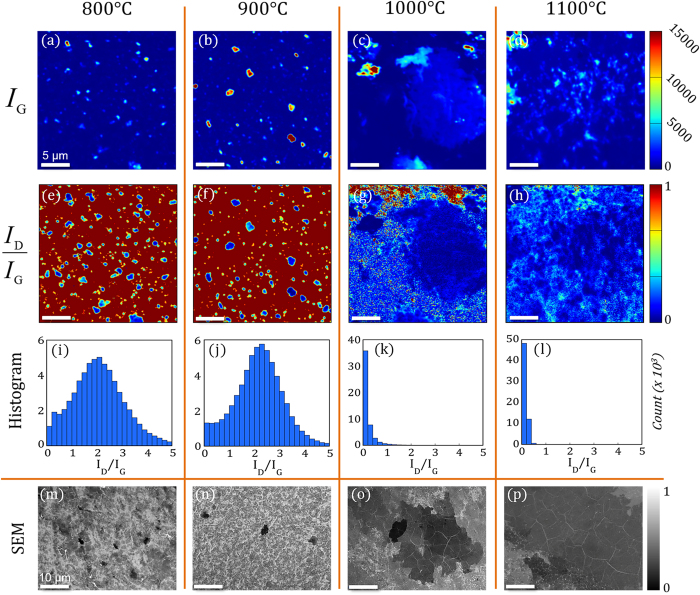
Characterization of the temperature dependent graphene growth morphologies. (**a**–**d**) Raman integrated intensity maps of the graphitic, G, phonon mode (I_G_). Regions with significant I_G_ values represent the presence of graphitic growth resolvable by the laser probe. A large graphene patch encompasses the Raman map obtained from the sample deposited at 1100 °C. All Raman maps are obtained from 25 μm × 25 μm areas. (**e**–**h**) Raman integrated intensity ratio maps of the D-peak relative to G-peak (I_D_/I_G_). The I_D_/I_G_ maps reveal regions of high quality graphene growth (i.e. a reduction in relative number of defects). (**i**–**l**) Histograms of the I_D_/I_G_ integrated Raman map raw data quantitate the transition to high quality graphene (ratios < 0.5) at deposition temperatures above 1000 °C. Counts for each I_D_/I_G_ bin (0.2 in size) are three orders of magnitude (Count x10^3^) higher than the scale bar suggests. (**m**–**p**) Representative SEM images of graphene films covering the nickel surface after deposition at each respective deposition temperature. The drastic increase in the size of graphene patches with constant layer thicknesses above 1000 °C is definitive, and for deposition of 1100 °C, distinct regions up to 100 μm in diameter were observed.

PVD graphene films of varying morphology are presented with representative integrated intensity Raman maps of the graphitic G peak (I_G_) in Fig. 3a–d, and scanning electron microscopy (SEM) images in Fig. 3m–p. Both, analytical techniques, Raman maps and SEM, corroborate an increase in the size of homogenous graphene regions as the deposition temperature is increased. Under the conditions presented here (see methods section), we attribute areas of greater SEM intensity to less graphene layers^47^. For SEM imaging on conducting substrates, electron-beam induced current and differential surface charging do not impact SEM contrast and, thus, contrast is derived directly from secondary electrons, generated by the substrate, attenuated by graphene layers^48^. SEM contrast analysis (not shown here) demonstrates similar layer thicknesses for both the 1000 °C and 1100 °C deposition temperatures, although a few marginally thicker regions were sparsely found at the 1000 °C deposition temperature. At the lower deposition temperatures, 800 °C and 900 °C, thick graphene patches with lateral dimensions on the order of a few microns were detected. As the deposition temperature increases to 1000 °C and 1100 °C, regions of constant thickness drastically increase in size with homogenous regions up to 100 μm in diameter observed from the 1100 °C deposition temperature. In conjunction with an increase in the size of distinct graphene regions, histograms of the integrated intensity ratio of the Raman D peak to the G peak (Fig. 3i–l) reveal a dramatic decrease in the quantity of defects relative to the graphitic signal at higher deposition temperatures. For the 1000 °C and 1100 °C deposition temperatures, histograms of the I_D_/I_G_ ratios (Fig. 3k,l) demonstrate the distribution of relative defects is predominantly less than 0.2, characteristic of high-quality graphene^49^. The I_D_/I_G_ histograms from the 800 °C and 900 °C deposition temperatures show a bell-shaped distribution with mean values < 2.0. Using eq. (1), the average nanocrystallite size was calculated as 9.2 nm and 8.4 nm at depositions temperatures of 800 °C and 900 °C, respectively,


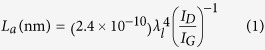


where L_*a*_ is the diameter of the crystallite size, λ_*l*_ is the wavelength of the Raman laser and I_D_/I_G_ is the ratio of the integrated intensities of the D and G peak^50^,^51^. It is apparent, from the I_D_/I_G_ Raman spectral maps in Fig. 3e,f, that regions of distinct graphene regions are surrounded by nanocrystalline graphene at lower deposition temperatures, while at higher deposition temperatures, large graphene patches are the predominant growth mode. The prominent transition from nanocrystalline graphene to large areas of constant graphene layer thickness, concomitant with a decrease in defects, limits our attention to as-grown PVD graphene deposited at higher temperatures.

**Figure 4:**
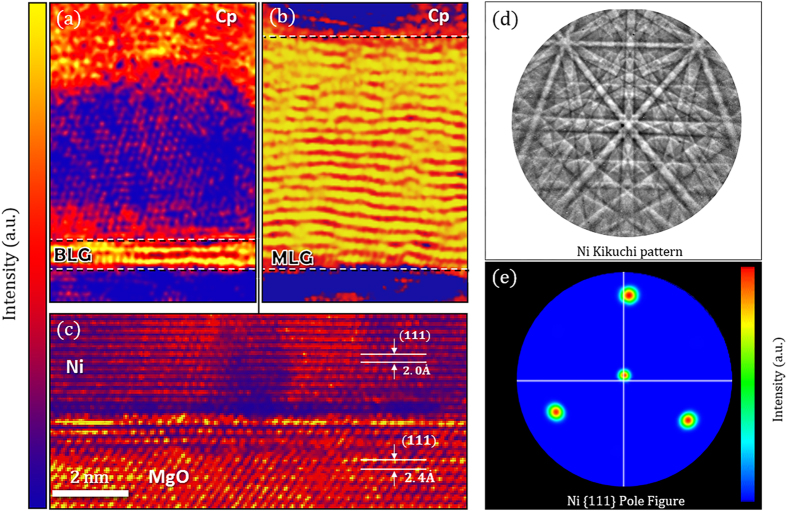
Cross-sectional TEM images of the | Magnesium Oxide (MgO) | Nickel (Ni) | Graphene (Gr) | heterostructure grown at 1100 °C with a protective capping layer (Cp) used for focused ion beam lift-out (colored to enhance contrast). (**a**) A representative cross-sectional area where bilayer graphene lattice fringes were observed (yellow). (**b**) An accompanied cross-sectional TEM image portraying multiple layers of graphene (MLG). BLG and MLG were the only observed growth modes from regions that express turbostratic Raman signatures. (**c**) The MgO – nickel substrate on which the graphene layers were grown. The respective distances between the Ni and MgO crystal planes were 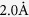 and 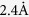 as expected for growth along the [111] direction. (**d**) The Ni Kikuchi pattern obtained from electron back-scattered diffraction (EBSD) demonstrating the high quality crystal growth. (**e**) An EBSD pole figure taken from a 250 × 150 um area showing the {111} crystal orientation.

To quantitatively determine the number of graphene layers grown via PVD and investigate the quality of the underlying heteroepitaxial nickel thin film, complementary TEM images were taken. Figure 4a,b displays cross-sectional TEM images from regions of as-grown graphene deposited at 1100 °C. The brighter lattice fringes (yellow) result from an increase in electron beam transmittance (i.e. intensity) due to the lower atomic density of carbon relative to the underlying nickel. Figure 4a identifies BLG while Fig. 4b displays MLG. Figure 4c–e confirms the single-crystalline nature of the nickel thin film is maintained after graphene growth with TEM imaging and electron back-scattered diffraction. From Fig. 4c, the atomic planes of the crystalline nickel mirrors the orientation of the MgO crystal where the respective 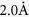 and 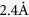 interplanar distances correspond to the expected (111) crystal planes. Furthermore, electron back-scattered diffraction (EBSD) identifies the Ni crystal orientation over larger areas. Figure 4d shows a representative Kikuchi pattern obtained from the EBSD analysis where the high quality crystal growth is evident by the clear indices and Kikuchi bands found in the 1100 °C Ni film (see the methods section for more details). The pole figure shown in Fig. 4e demonstrates the single {111} Ni crystal orientation taken at steps of 290 nm, probed for a total area of over 5 square mm. Finer maps with steps of 29 nm were also taken to ensure the microstructure was not varying at smaller scales. Importantly, these cross-sectional areas were obtained from graphene regions exhibiting key turbostratic Raman signatures detailed in subsequent sections and are representative of PVD graphene growth after carbon deposition at 1000 °C and 1100 °C. The Raman selection rule renders the Raman signal from SLG absent on nickel^25^,^52^ and SLG was not observed from any of the cross-sectional samples examined. This allows for a detailed analysis of characteristic turbostratic Raman signatures by deconvoluting features that could be attributed to both single-layer and turbostratic graphene as a result of their similar electronic structure.

### Characteristic Raman bands for PVD graphene growth

Raman spectroscopy probes the electronic and vibrational properties of molecules and crystalline materials through inelastic scattering of photons by phonons and is a powerful tool for graphene characterization^40^. In brief, the prominent Raman active phonon modes of graphene are known as the D, G and 2D (also known as G’^53^) bands. The 2D mode is an overtone of the in-plane transverse optic (iTO) branch and is observed with a Raman Stokes shift of 2700 cm^−1^. It displays a single Lorentzian profile for SLG, while for AB Bernal MLG, the 2D mode splits into multiple components, broadening its shape as a result of the altered band structure and dispersion near the Fermi level^22^,^53^. Overall, the presence, position, full width at half maximum (FWHM), integrated intensity (*I*) and line-shape of Raman spectral features provide quantitative and qualitative information about the structure and properties of graphene^40^, including the interlayer coupling, which is significant in the context of this study^30^,^31^,^35^,^54^,^55^,^56^,^57^. For more detailed information regarding the characterization of graphene via Raman spectroscopy, we refer the reader to several insightful reviews^25^,^40^,^49^,^51^,^58^,^59^,^60^.

**Figure 5:**
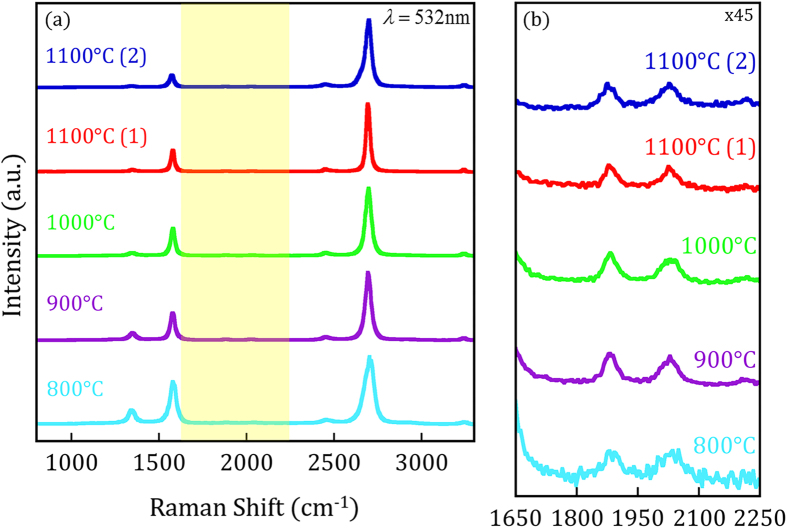
Normalized Raman spectra raw data using a laser excitation energy of E_L_ = 2.33 eV, obtained for growth temperatures 800–1100 °C. The spectra taken from the 1100 °C sample was acquired from the corresponding colored markers shown in Fig. 8a. (**a**) Raman spectra indicating the high quality graphene growth through PVD. The reduction in D-peak intensity as deposition temperature increases agrees with the I_D_/I_G_ Raman maps and SEM images presented in Fig. 3. The area highlighted in yellow signifies the location of combination Raman modes magnified in (**b**). The peak shape, intensity, and location of the TS_1_ and TS_2_ combination Raman modes are indicative of turbostratic stacking.

Figure 5a displays normalized Raman spectra of as-grown PVD graphene from each respective deposition temperature. The data for the samples deposited at 1100 °C were obtained from the areas indicated with a colored marker in the 2D Raman maps of Fig. 8a. The G peak is primarily a response to an in-plane phonon mode found in graphitic carbon where its intensity with respect to the 2D peak is often used to qualify the number of AB stacked graphene layers^53^. The D peak is active in the presence of structural defects or graphene edges. The normalized Raman spectra presented in Fig. 5 demonstrate the high-quality growth of graphene on nickel through PVD and, in agreement with Fig. 3, exhibit a reduction in the relative D-peak intensity as deposition temperature increases. Specific values for the spectra in Fig. 5 have been provided in Table S1 for reference. A detailed examination of the 2D peak features can provide further details of the graphene growth due to its sensitivity to the electronic and phonon band structure^40^,^59^.

**Figure 6:**
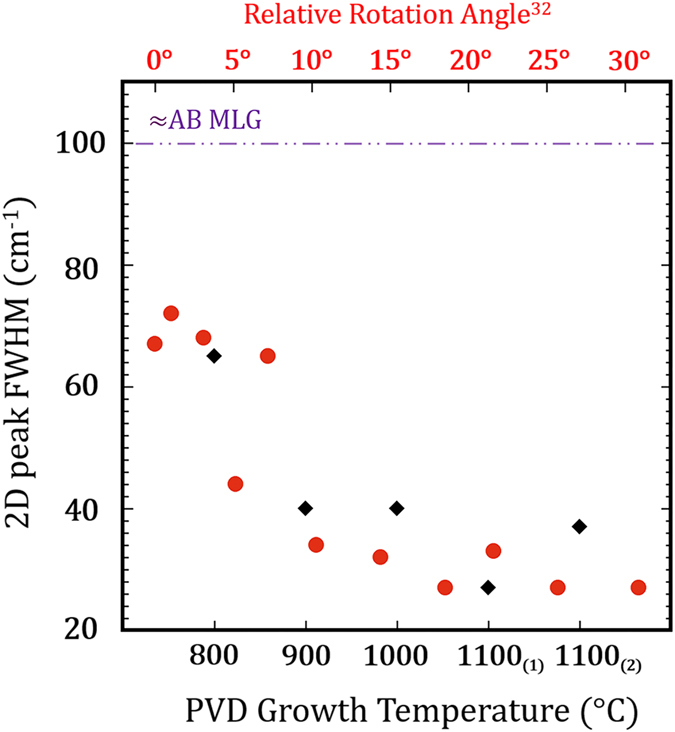
The 2D full width at half maximum (black markers) of the Raman spectra presented in Fig. 5 as a function of deposition temperature. The red markers were adapted from Kim *et al.*^32^ showing the 2D FHWM as a function of the rotation angle for twisted BLG. The 2D FWHM for turbostratic PVD graphene trends downward towards the expected values for SLG as the deposition temperature increases. At high deposition temperatures, 2D FWHM values as low as 27 cm^−1^ may suggest high relative rotation angles. The purple dashed line indicates the expected 2D FWHM for Bernal stacked graphene layers.

The normalized Raman spectra, shown in Fig. 5a, reveal a 2D peak intensity significantly greater than the G peak intensity for PVD deposition temperatures above 800 °C, consistent with turbostratic graphene. The profile of the 2D phonon mode of turbostratic graphene portrays that of SLG and, therefore, is composed of a single Lorentz peak resulting from the linear band dispersion of the weakly coupled layers^24^,^25^,^32^,^40^,^61^. Turbostratic graphene has an active Raman signal on nickel^25^, despite its similar electronic structure to SLG. The 2D FWHM of turbostratic graphene is distinct from that of ordered AB Bernal MLG, which successively broadens as layer numbers increase^40^,^53^,^59^. The 2D peak, from samples deposited above 800 °C, fit well to a single Lorentzian and their observed FWHM are narrower than would be expected for AB MLG. Figure 6 presents the 2D peak FWHM from the spectra presented in Fig. 5a along with the reported 2D FWHM for rotated graphene bilayers by Kim *et al.*^32^. The 2D FWHM from all samples presented in Fig. 5a fall within the range of the reported rotated graphene bilayers with a 2D FWHM as low as 27 cm^−1^, observed from the 1100 °C(1) spectra. A 2D FWHM of this magnitude is on the order of the 2D FWHM reported for SLG^53^ as well as for twisted BLG with high angle layer rotations^32^,^35^. Interestingly, the plot suggests the relative rotation between graphene layers increases as deposition temperature increases.

### Combination Raman Modes

Rao *et al.*^30^ and Cong *et al.*^31^ have reported combination Raman modes between 1650 cm^−1^ and 2300 cm^−1^ differ in intensity and position for SLG, turbostratic (*i.e.* incommensurate) and Bernal phase (*i.e.* commensurate) MLG. Of particular interest are the iTALO^−^, iTALO^+^, iTOLA, and LOLA modes that are distinct for turbostratic graphene and become convoluted for AB Bernal stacked graphene layers. The iTALO^−^, iTALO^+^, iTOLA and LOLA modes are believed to arise from the combination of in-plane transverse acoustic (iTA) and the longitudinal optic (LO), iTA and longitudinal acoustic (LA) and LO + LA modes. Here, we designate the iTALO^−^ mode as TS_1_ and the iTOLA/LOLA modes as TS_2_. Additionally, the iTOTA, R and R’ Raman phonon modes, can also be used to classify interlayer coupling^30^ and the relative layer rotation angle between graphene layers^33^,^34^,^35^,^62^. While these peaks were detected within turbostratic regions of our samples, their presence was inconsistent.

**Figure 7:**
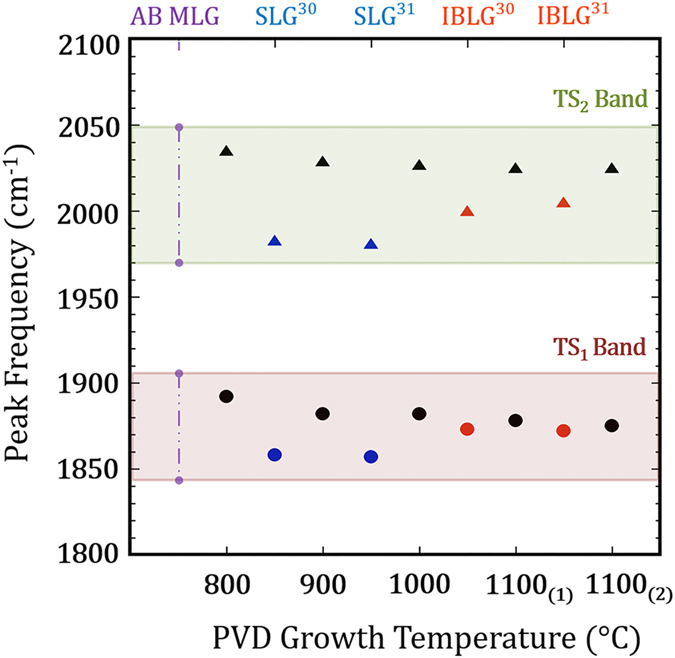
Combination Raman modes of turbostratic graphene compared to reported literature values. The TS_1_ (circles) & TS_2_ (triangles) peak positions for different PVD deposition temperatures, shown with black markers. The SLG (blue) and IBLG (red) where added from Rao *et al.*^30^ and Cong *et al.*^31^ for reference. The peak positions of TS_1_ and TS_2_ are blue-shifted from the SLG and IBLG values. At 1100 °C growth temperatures, the TS_1_ peak nearly matches the position observed from IBLG while the TS_2_ peak remains blue-shifted from IBLG peak positions. The purple vertical dashed line represents the absence of prominent peaks for AB Bernal MLG.

Figure 5b magnifies the frequency range where combination Raman modes reside and presents the same spectra shown in Fig. 5a. For turbostratic graphene, the TS_1_ and TS_2_ modes are well defined with distinct intensities while these peaks disperse into the background for AB Bernal BLG and MLG. Further, turbostratic graphene exhibits greater TS_1_ and TS_2_ peak intensities than SLG, due to stiffening of the phonon modes and, importantly, their peak positions are blue-shifted^30^,^31^. The TS_1_ and TS_2_ peaks are clearly observed in Fig. 5b with distinct intensity from as-grown samples deposited at 900 °C, 1000 °C and 1100 °C. The TS_1_ (circles) and TS_2_ (triangles) peak positions are displayed for all deposition temperatures in Fig. 7. To serve as a reference, the reported TS peak positions for SLG (blue markers) and incommensurate BLG (IBLG) (red markers) have also been added from Rao *et al.*^30^ and Cong *et al.*^31^. The purple line in Fig. 7, labeled AB MLG, is used to represent the absence of clear TS peaks from AB Bernal graphene layers. The TS_1_ and TS_2_ peak frequencies are blue-shifted for all deposition temperatures and the magnitude of their blue-shift is reduced as deposition temperature is increased (Fig. 7). Moreover, the turbostratic graphene TS_1_ peak frequencies shown here closely resemble the reported TS_1_ peak positions of IBLG (*i.e.* turbostratic BLG) while the turbostratic TS_2_ peaks are upshifted from reported IBLG TS_2_ peak frequencies. This observation is clear when examining the 1100 °C samples; the TS_1_ peak position is within 2 cm^−1^ of the reported IBLG values while the TS_2_ peak position is found at higher frequencies. The increased blue-shift seen in the TS_2_ peak is consistent across all deposition temperatures and could result from an intrinsic difference between the phonon modes of IBLG and as-grown turbostratic graphene investigated here. Finally, the M band^30^,^31^, which is an overtone of the oTO mode within the wavelength 1650–1750 cm^−1^ that is present for Bernal phase BLG and MLG, was not detected in our PVD graphene, further supporting the layer rotations conclusion. The presence of as-grown PVD turbostratic graphene is systematically established with features of the 2D peak, combination Raman modes, in conjunction with high-resolution TEM analysis, our focus proceeds to mapping turbostratic graphene with pertinent Raman signatures.

### Mapping Turbostratic Graphene

**Figure 8:**
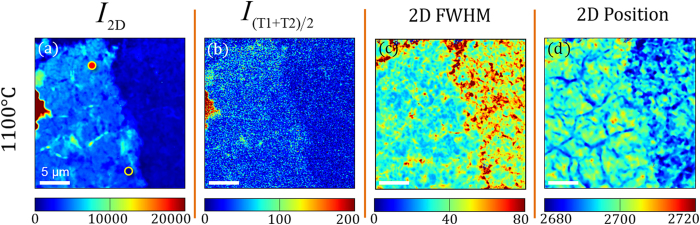
Characteristic Raman maps demonstrating the prevalence of turbostratic graphene over a 25 μm × 25 μm area from a sample deposited at 1100 °C. The figure displays Raman maps of the (**a**) integrated 2D peak intensity, (**b**) average integrated intensity of the TS peaks, (**c**) 2D full width at half maximum (FWHM) and (**d**) frequency of the 2D peak. The region of increased 2D integrated intensity, located on the left-half of the figure, spatially corresponds with the presence of combination Raman modes, narrower 2D peak FWHM and an upshift in 2D peak frequency. Color markers present in Fig. 8a indicate where spectra of the same line colors were acquired in Fig. 5.

The Raman maps of the integrated intensity of the 2D peak (I_2D_), averaged TS peaks (I_(TS_1_+TS_2_)/2_), 2D FWHM and 2D peak frequency, shown in Fig. 8, outline the prevalence of turbostratic graphene on Ni(111) deposited at 1100 °C. High 2D integrated intensity values suggest the absence of AB Bernal stacking, supporting the presence of MLG with properties similar to SLG. The I_2D_ Raman map (Fig. 8a) shows a clear transition in 2D intensity from left (*I* >5000) to right (*I* <5000). Traditionally, higher 2D intensities would be associated to SLG^53^, but as explained in the previous section, we attribute the increased intensity to the decoupled nature of turbostratic graphene. A 2D FWHM (Fig. 8c) on the order of 30 cm^−1^ is observed over the area of interest, indicating this region preserves the electronic structure of SLG^26^,^61^. In addition, upshifted 2D peak frequencies (Fig. 8d), indicative of layer rotations^32^,^59^,^63^,^64^ found in turbostratic graphene, follow the intensity profile of the I_2D_ and 2D FWHM Raman maps which are direct evidence of single Lorentzian Raman peaks and turbostratic graphene^25^,^40^. Intriguingly, the Raman map of the 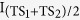 intensity (Fig. 8b) closely mirrors the I_2D_, 2D FWHM and 2D peak position profiles. These observations establish the TS peaks as signatures that can be used to spatially map graphene with incommensurate stacking, despite their limited intensity. The regions that exhibit high I_2D_, 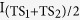 values, along with narrow 2D peak widths and an upshifted peak frequency, comprise a substantial area of the 25 μm × 25 μm Raman maps in Fig. 8 and depict domain-like growth of turbostratic graphene on Ni(111). These Raman maps demonstrate the large-area coverage of turbostratic graphene after carbon deposition at 1100 °C and exemplify the inherent differences between graphene growth on nickel and graphene grown on copper, where the prevalence of turbostratic graphene is sparse^36^. The spatial identification of large-area turbostratic graphene grown via PVD demonstrated here could be ideal for device architectures that would leverage the properties of SLG in a multi-layered structure.

## Conclusion

Using physical vapor deposition (PVD), large-area turbostratic graphene was grown on heteroepitaxial Ni(111) thin films in a continual process with precise control of the deposition temperature, beam flux, and total amount of carbon deposited. SEM images and Raman maps reveal a substantial increase in graphene regions of constant thickness at higher deposition temperatures. Distinct graphene regions of up to 100 μm in diameter were observed at carbon deposition temperatures of 1100 °C. Correspondingly, low I_D_/I_G_ ratios observed from as-grown samples deposited at 1000 °C and 1100 °C represent the high-quality nature of the graphene grown via PVD. High-resolution TEM imaging of focused ion beam prepared cross-sections directly identify bilayer and multilayer graphene (MLG) growth regimes in areas that exhibited characteristic turbostratic graphene Raman signatures. The characteristics of the 2D Raman mode and combination iTALO^−^/iTALO^+^ (TS_1_) and iTOLA/LOLA (TS_2_) Raman modes were employed to investigate interlayer coupling between as-grown MLG layers. Turbostratic graphene was identified using distinct peak intensities and blue-shifted frequencies of combination Raman modes accompanied by characteristic single Lorentzian 2D peak profiles and direct layer quantification through TEM analysis. Raman maps of turbostratic graphene Raman modes reveal significant coverage of turbostratic graphene on the Ni(111) at high deposition temperatures using PVD. The spatial identification of large-area turbostratic graphene using PVD on nickel thin films provides a unique opportunity to explore the fundamental properties and viability of turbostratic graphene for future applications such as data storage and transmission technologies.

## Experimental Methods

### Sample Preparation

The growth method present here has been adapted from Iwasaki *et al.*^39^ Heteroepitaxial nickel(Ni) thin films were grown via high-vacuum electron-beam evaporation at base pressures of 2 × 10^−9^ Torr from a 99.999% Ni source on single crystalline magnesium oxide (MgO(111)) substrates. Due to the difference in lattice parameters between Ni(111) and MgO(111), 100 nm of nickel was deposited at 300 °C to promote single domain growth and decrease the surface roughness of the thin film^38^. Subsequently, an additional 100 nm of nickel was deposited at 600 °C to increase the nickel grain size^39^. Following growth of the Ni(111) substrates, carbon was deposited at a rate of less than 0.5 Å/s through sublimation of a highly oriented pyrolytic graphite source at sample deposition temperatures of 800 °C, 900 °C 1000 °C, and 1100 °C. We note, nickel thin films remained continuous with no agglomeration for all deposition temperatures. At high temperature, dissolved carbon segregates on the nickel surface below the solubility limit then readily nucleates once saturation is reached - either through precipitation or as the result of adatoms impinging on the surface^43^. Carbon segregation and precipitation curves for 200 nm thick Ni thin films can be found in Figure S1. After carbon deposition, samples were cooled to 550 °C and maintained at this temperature for 2 hours to heal the nickel film^17^.

### Raman, SEM, and TEM Characterization

The Raman spectra and Raman maps were taken using a Witec Confocal Raman Microscope Alpha 300R using a 532 nm laser wavelength. The 25 μm × 25 μm Raman maps were constructed from 250 × 250 point spectra. The Raman maps for each deposition temperature were taken of the same region, respectively. For all integrated intensity maps, the 2D band was designated 2670–2730 cm^−1^, the G band was designated 1560–1600 cm^−1^, and the D band was designated 1325–1375 cm^−1^. The TS_1_ and TS_2_ peaks were designated 1860–1910 cm^−1^ and 2000–2050 cm^−1^, respectively. The peak intensity ratios, seen in Table S1, were calculated using the peak height after background subtraction. The bin size for all histograms is 0.2.

The SEM images were taken using a JEOL 7600F Analytical SEM at an acceleration voltage of 2 kV and a working distance of 3mm in order to excite secondary electrons from the graphene layers. The TEM images were taken using a double aberration corrected JEOL ARM 200F at an acceleration voltage of 80 kV to mitigate any possible beam damage. The EBSD data was taken at an acceleration voltage of 20 kV and incidence angle of 70°. The mapping of the crystalline orientation takes into account this 20° offset geometry from normal which is why the [111] index is just above the pattern shown in Fig. 4d.

## Additional Information

**How to cite this article**: Garlow, J. A. *et al.* Large-Area Growth of Turbostratic Graphene on Ni(111) via Physical Vapor Deposition. *Sci. Rep.* **6**, 19804; doi: 10.1038/srep19804 (2016).

## Supplementary Material

Supplementary Information

## Figures and Tables

**Figure 1:**
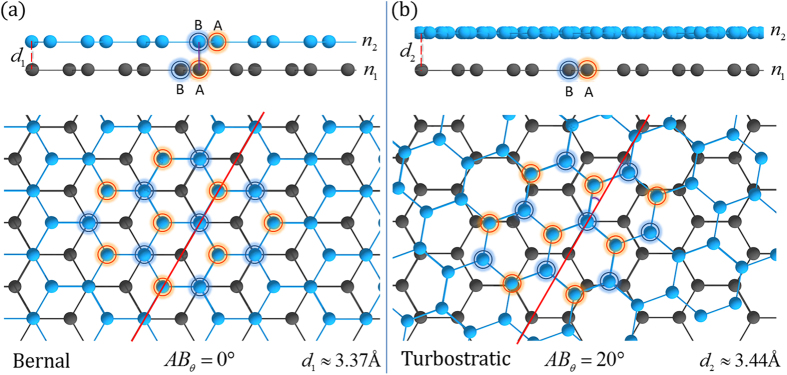
A representative illustration of bilayer graphene with different stacking orientations. (**a**)Bernal phase, or AB stacked, BLG refers to the stacking of the B site (highlighted navy rings) from the top light blue graphene layer *n*_*2*_, directly above the A site (highlighted orange rings) for the bottom grey graphene layer *n*_*1*_. This type of stacking has a relative site rotation of *AB* = 0° for the neighboring layers (*n*_*1*_, *n*_*2*_) resulting in orbital hybridization and an interplanar distance of 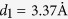. (**b**) Turbostratic graphene is a random relative rotation between adjacent layers, in this case *AB* = 20°, which mitigates the orbital hybridization and has an increased interplanar distance of 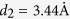. The coupling between the stacking orientations is different due to the orbital interactions and can be studied using characteristic Raman bands.
