# Supplementary material for: Large-Area Growth of Turbostratic Graphene on Ni(111) via Physical Vapor Deposition
Source: Sci Rep. 2016 Jan 29;6:19804. doi: 10.1038/srep19804 (PMC4731759; doi:10.1038/srep19804)
Supplement: Supplementary Information [file srep19804-s1.doc]

**Supporting Information**

for

Large-Area Growth of Turbostratic Graphene on Ni(111) via Physical Vapor Deposition

Joseph A. Garlow*^†,‡^*, Lawrence Barrett*^§^*, Lijun Wu^†^, Kim Kisslinger^⊥^, Yimei Zhu*^†,‡^**, and Javier F. Pulecio*^†^**

^†^Condensed Matter Physics and Material Science Department, Brookhaven National Laboratory, Upton, NY 11973, ^‡^ Material Science and Engineering Department, Stony Brook University, Stony Brook, NY 11794, *^§^* Department of Physics, Brigham Young University, Provo, UT 84602, ^⊥^ Center for Functional Nanomaterials, Brookhaven National Laboratory, Upton, NY 11973


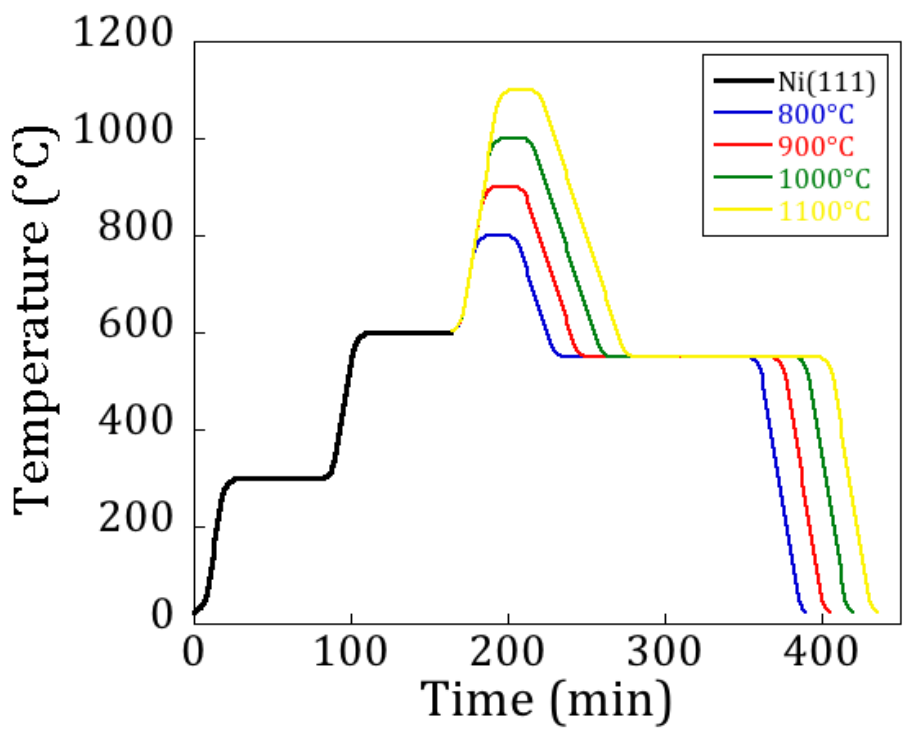

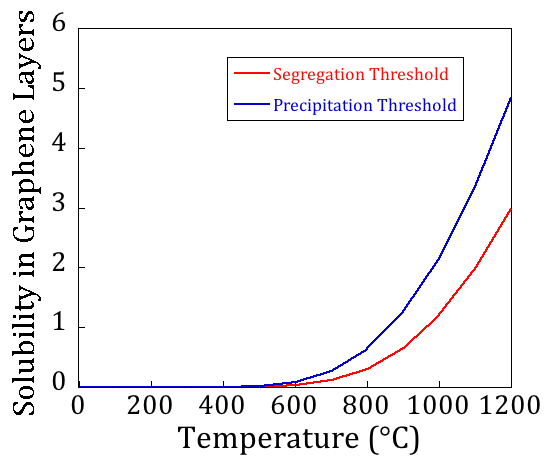


Figure S1: (a) Illustration of the Ni(111) thin film substrate and graphene growth process adapted from Iwasaki et al^1^. 100nm of nickel was deposited at 300°C and 600°C. Subsequently, carbon was deposited at temperatures 800°C, 900°C, 1000°C or 1100°C, without breaking vacuum. Samples were then cooled at a constant rate to 550°C, at which they were maintained for 2 hours before cooling to room temperature. (b) Carbon solubility in 200nm Ni thin films. Solubility equations were obtained from Baraton et al.^2^

1000°C

1100°C

800°C

900°C

1100°C

1000°C

Figure S2: Raman integrated intensity ratio maps of the D-peak relative to G-peak (I_D_/I_G_) re-scaled to that of the histograms in Figure 2i-l. The I_D_/I_G_ maps reveal the drastic increase in graphene quality at high deposition temperatures and provide more detail on the location of observed high I_D_/I_G_ values at the low deposition temperatures.


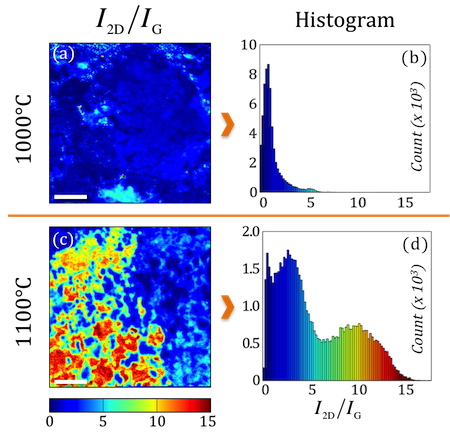


Figure S3: Analysis of graphene growth modes utilizing the 2D to G peak integrated intensity ratio (I_2D_/I_G_). (a,c) Raman maps of the I_2D_/I_G_ ratio qualitatively identify the location of turbostratic graphene. Fluctuations in the I_2D_/I_G_ map result from variations in the integrated G-peak intensity. (b,d) Histograms of the I_2D_/I_G_ Raman maps demonstrating the significant difference in graphene growth between the two deposition temperatures with a prominent peak for the 1100°C sample at I_2D_/I_G_ = 10.

Table S1: Raman characteristics of PVD graphene on Ni from the spectra presented in Figure 4. All ratios were calculated after background subtraction using the same spectral widths used for the Raman maps seen in Figure 7.

**References:**

1. Iwasaki, T. *et al.* Long-range ordered single-crystal graphene on high-quality heteroepitaxial Ni thin films grown on MgO(111). *Nano Lett.* **11,** 79–84 (2011).

2. Baraton, L. *et al.* On the mechanisms of precipitation of graphene on nickel thin films. *Europhysics Lett.* **96,** 46003 (2011).
